# Knowledge mapping analysis of mental health research on COVID-19

**DOI:** 10.3389/fpsyt.2022.931575

**Published:** 2022-08-31

**Authors:** Runjin Zhou, Xiaoling Lin, Jiamei Xu, Xingdong Lin, Zhibing Wu

**Affiliations:** ^1^Medical College of Acupuncture-Moxibustion and Rehabilitation, Guangzhou University of Chinese Medicine, Guangzhou, China; ^2^The First Clinical Medical College of Guangzhou University of Chinese Medicine, Guangzhou, China; ^3^The First Affiliated Hospital of Guangzhou University of Chinese Medicine, Guangzhou, China; ^4^The Third Affiliated Hospital of Guangzhou University of Chinese Medicine, Guangzhou, China

**Keywords:** mental health, COVID-19, VOSviewer, CiteSpace, scientometric analysis

## Abstract

**Objective:**

A bibliometric analysis of COVID-19 is conducted to examine the developmental context, research hotspots, and frontiers of mental health.

**Methods:**

Using the Web of Science Core Collection (WOSCC), we have retrieved articles on mental health research related to COVID-19 which were published between 2019 and 2021. The coauthorship of countries, institutes, and authors was analyzed using VOSviewer 1.6.17, and the co-citation map of authors/references was analyzed as well. CiteSpace version 5.8.R3 was used to analyze keyword clusters and forecast research frontiers.

**Results:**

There were 8,856 articles retrieved, including 10,559 research institutes and 1,407 academic journals. The most published country and institutes were the United States (2190) and the University of London (373). Wang, Chengyu owned the highest co-citations (1810). Frontier topics can be identified by trending keywords, including “anxiety,” “depression,” “psychological distress,” “quarantine,” “post-traumatic stress disorder (PTSD),” “insomnia,” and “Healthcare workers.”

**Conclusion:**

The most common psychological problems of people during the epidemic are anxiety and depression. Insomnia and PTSD need to be solved under the normalization of the epidemic. GAD-7 and PHQ-9 scales are the most convenient and effective for screening anxiety and depression. Healthcare workers, older adults, and college students should be concerned, and social and family support is essential.

## Introduction

Since December 2019, the novel coronavirus pneumonia (COVID-19) has spread throughout the world, and the number of confirmed clinical cases worldwide is still on the rise. With the development and vaccination of vaccines, the global epidemic has been brought under control to a certain extent. However, with the continuous emergence of mutant strains (1, 2), nucleic acid testing and quarantine observation are still the main means of epidemic prevention and control. When such sudden public health events occur, people will have a large number of psychological problems, including anxiety and post-traumatic stress disorder (PTSD) (3). Over the past 2 years, more and more research has been performed on mental health. Therefore, studying the impact of COVID-19 on public mental health is of great significance for timely and effective interventions in the future. At present, it is necessary to sort out the developmental history of this research field scientifically and systematically and to further present its future research hotspots and developmental direction.

After searching the relevant article database, it was found that there were few visual articles on mental research with COVID-19. Software such as CiteSpace and VOSviewer analyze citations visually under the background of scientometrics and data visualization. In this study, CiteSpace and VOSviewer software were used to generate visual knowledge maps of article data about mental health research under COVID-19 on the Web of Science, attempting to provide research ideas for clinical and basic research workers.

## Materials and methods

### Data source and search strategy

On 27 February 2022, articles were retrieved online using the Science Citation Index-Expanded (SCI-E) of the Web of Science Core Collection (WOSCC). The strategy for retrieving the data consisted of TS = (COVID-19 OR 2019-nCOV OR SARS-CoV-2 OR novel coronavirus pneumonia OR coronavirus disease-2019) AND TS = (psychology OR psychological OR psychological health OR psycholog^*^ OR mentality OR mental^*^ OR mental health), index=SCI-EXPANDED, and time span: 2019-12-01 to 2021-12-31. We only selected articles as the document type, and the language was limited to English.

### Data collection

Two research members (RJZ and XLL) downloaded raw data from WOSCC, which was then imported into VOSviewer 1.6.17 and CiteSpace version 5.8.R3, and the information generated by the software was imported into Excel 2007.

### Statistical methods

The data from the WOSCC database were analyzed, including countries/regions, institutes, authors, journal sources, number of citations/articles, and impact factor.

VOSviewer is a bibliometric analysis software jointly created by Nees Jan van Eck and Ludo Waltman to map scientific knowledge (4). We performed a co-occurrence analysis of countries, institutions, and authors (**Figure 2**) using VOSviewer. Similarly, co-citation maps of references and authors were generated using VOSviewer (**Figure 4**). There were three types of mapping generated, namely, network visualization, overlay visualization, and density visualization. In network visualization, elements of the same color were clustered together, so kinds of colors represented different clusters. Within the overlay visualization, it generated a timeline at the bottom right corner, displaying blue for older articles and darker yellow for more recent ones. For density visualization, the larger and deeper the yellow element, the greater its influence on the field.

CiteSpace is a tool for visualizing and analyzing trends and patterns in scientific papers (5, 6). It was used to analyze keywords and keywords with the strongest citation bursts (**Figures 5**, **6**). A node in the co-citation knowledge graph represents a document, and the size of the node is proportional to the number of references cited in a particular period of time. Moreover, the strength value of the keyword bursts indicates its hotspot research (**Figure 7**). As a result, it will be easier to observe the trend of various research hotspots over time (7).

## Results

### Annual articles and citations

A total of 8,856 articles were included from 2020 (*n* = 2,207) to 2021 (*n* = 6,649), and the citations of these articles also increased dramatically from 2020 (*n* = 14,404) to 2021 (*n* = 89,099), with a total of 103,503 citations.

### Distribution of journals and cited articles

**Figure 1:**
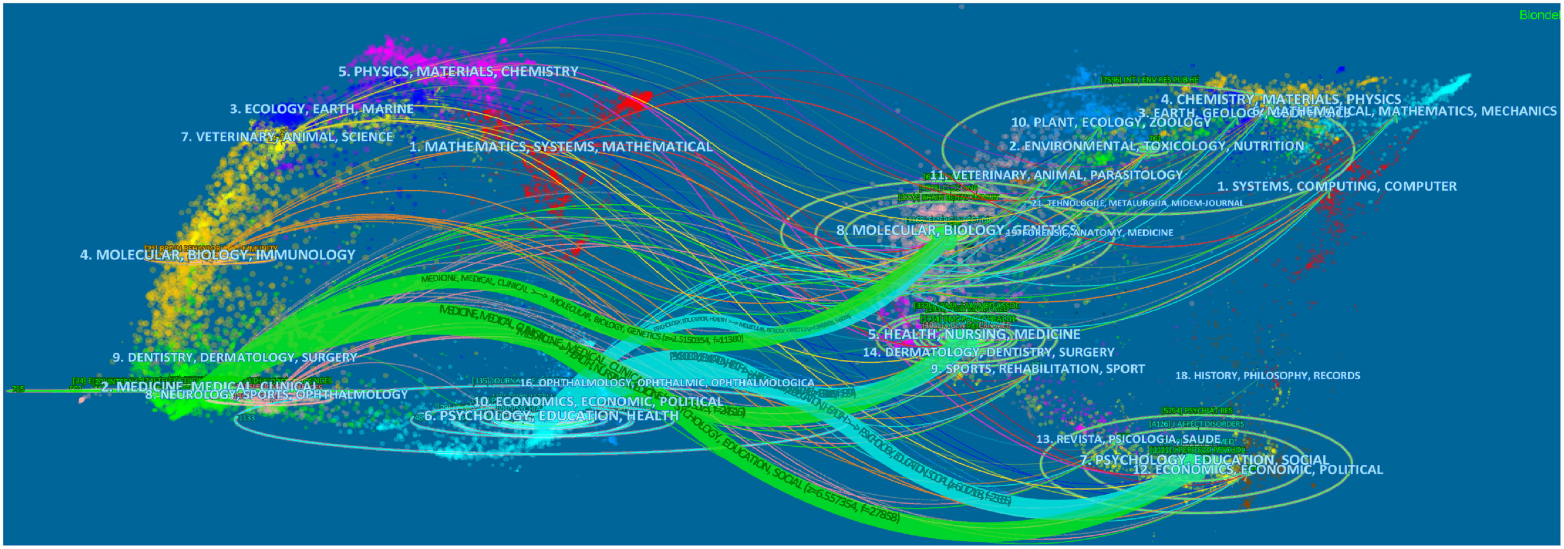
The dual-map overlay of journals on mental health research related to COVID-19.

A total of 1,407 academic journals have published articles on mental health related to COVID-19. Table 1 lists the top 10 journals with a total of 2,741 articles, accounting for 30.95% of the total number of articles. *International Journal of Environmental Research and Public Health* published the most articles (899 articles, 10.15%), followed by the Journal of *Frontiers in Psychiatry* (459 articles, 5.18%) and *PLOS ONE* (362 articles, 4.09%). Dual-map overlay of journals is shown in Figure 1, where the left side corresponds to the citation map and the right side corresponds to the cited journal map (8). Lines on the map start from the left to the right, representing citation links for the disciplines covered by the journal. There were six citation paths, which were roughly divided into two colors. For the upper path, medicine/medical/clinical journals represented by green and psychology/education/health journals represented by light blue are cited in molecular/biology/genetics journals. The middle path, papers published in medicine/medical/clinical journals represented by green, and psychology/education/health journals represented by light blue are cited in health/nursing/medicine area. The bottom path, papers published in medicine/medical/clinical journals represented by green, and psychology/education/health journals represented by light blue are cited in the psychology/education/social area. Table 2 shows the 10 most frequently cited articles.

**Table 1:** The top 10 journals that published articles on mental health research related to COVID-19.

**Rank**	**Journal**	**Country**	**Count**	**IF 2021**
1	International Journal of Environmental Research and Public Health	Switzerland	899	3.390
2	Frontiers in Psychiatry	United States	459	4.157
3	PLOS ONE	United States	362	3.240
4	Frontiers in Public Health	Switzerland	203	3.709
5	BMJ Open	England	188	2.692
6	Psychiatry Research	Netherlands	140	3.222
7	Journal of Affective Disorders	Netherlands	137	4.839
8	Journal of Medical Internet Research	Canada	122	5.428
9	Sustainability	Switzerland	120	3.251
10	Healthcare	Switzerland	111	2.645

**Table 2:** Top 10 most cited articles on mental health research related to COVID-19.

**Rank**	**First Author**	**Title**	**Journal**	**Cited**	**Highlight**
1	Lai, Jianbo	Factors Associated with Mental Health Outcomes Among Health Care Workers Exposed to Coronavirus Disease 2019	JAMA Network Open	2,642	Multivariable logistic regression analysis was performed to identify factors associated with mental health outcomes among health care workers in China. The degree of symptoms of depression, anxiety, insomnia and distress was assessed.
2	Wang, Cuiyan	Immediate Psychological Responses and Associated Factors during the Initial Stage of the 2019 Coronavirus Disease (COVID-19) Epidemic among the General Population in China	International Journal of Environmental	2,373	The study was to survey the general public in China to better understand their levels of psychological impact, anxiety, depression, and stress during the initial stage of the COVID-19 outbreak. The data will be used for future reference
3	Holmes, Emily A	Multidisciplinary research priorities for the COVID-19 pandemic: a call for action for mental health science	Lancet Psychiatry	1,852	Researchers explored the psychological, social, and neuroscientific effects of COVID-19 in UK and set out the immediate priorities and longer-term strategies for mental health science research. Emphasis on mitigating mental health consequences for vulnerable groups
4	Cao, Wenjun	The psychological impact of the COVID-19 epidemic on college students in China	Psychiatry Research	1,448	Researchers sampled college students from Changzhi medical college in China by using cluster sampling. results showed that economic effects, effects on daily life and delays in academic activities, were positively associated with anxiety symptoms, social support was negatively correlated with the level of anxiety
5	Van Bavel, Jay J	Using social and behavioral science to support COVID-19 pandemic response	Nature Human Behavior	1,361	The study discussed evidence from a selection of research topics relevant to pandemics. To help align human behavior with the recommendations of epidemiologists and public health experts
6	Huang, Yeen	Generalized anxiety disorder, depressive symptoms and sleep quality during COVID-19 outbreak in China: a web-based cross-sectional survey	Psychiatry Research	1,144	Using a web-based cross-sectional survey to assess the mental health burden of Chinese public. results suggested Younger people, people spending too much time thinking about the outbreak, and healthcare workers were at high risk of mental illness
7	Wang, Cuiyan	A longitudinal study on the mental health of general population during the COVID-19 epidemic in China	Brain Behavior and Immunity	928	This longitudinal study surveyed the general population twice - during the initial outbreak, and the epidemic's peak four weeks later, surveying demographics, symptoms, knowledge, concerns, and precautionary measures against COVID-19
8	Zhong, BaoLiang	Knowledge, attitudes, and practices toward COVID-19 among Chinese residents during the rapid rise period of the COVID-19 outbreak: a quick online cross-sectional survey	International Journal of Biological Sciences	857	The study investigated Chinese residents' knowledge, attitudes, and practices (KAP) during the rapid rise period of the outbreak. Most Chinese residents of a relatively high socioeconomic status were knowledgeable about COVID-19, hold optimistic attitudes, and have appropriate practices toward it
9	Czeisler, Mark E	Mental Health, Substance Use, and Suicidal Ideation During the COVID-19 Pandemic - United States, June 24–30, 2020	MMWR-Morbidity and Mortality Weekly Report	629	The study assessed mental health, substance use, and suicidal ideation during the pandemic, representative panel surveys were conducted among adults aged ≥18 years across the United States
10	Mazza, Cristina	A Nationwide Survey of Psychological Distress among Italian People during the COVID-19 Pandemic: Immediate Psychological Responses and Associated Factors	International Journal of Environmental Research and Public Health	637	The study aimed to establish the prevalence of psychiatric symptoms and identify risk and protective factors for psychological distress among Italians. Results showed that Having an acquaintance infected was associated with increased levels of both depression and stress, those with a family member infected and young person who had to work outside their domicile presented higher levels of anxiety and stress, respectively

### Distribution of countries and institutes

**Figure 2:**
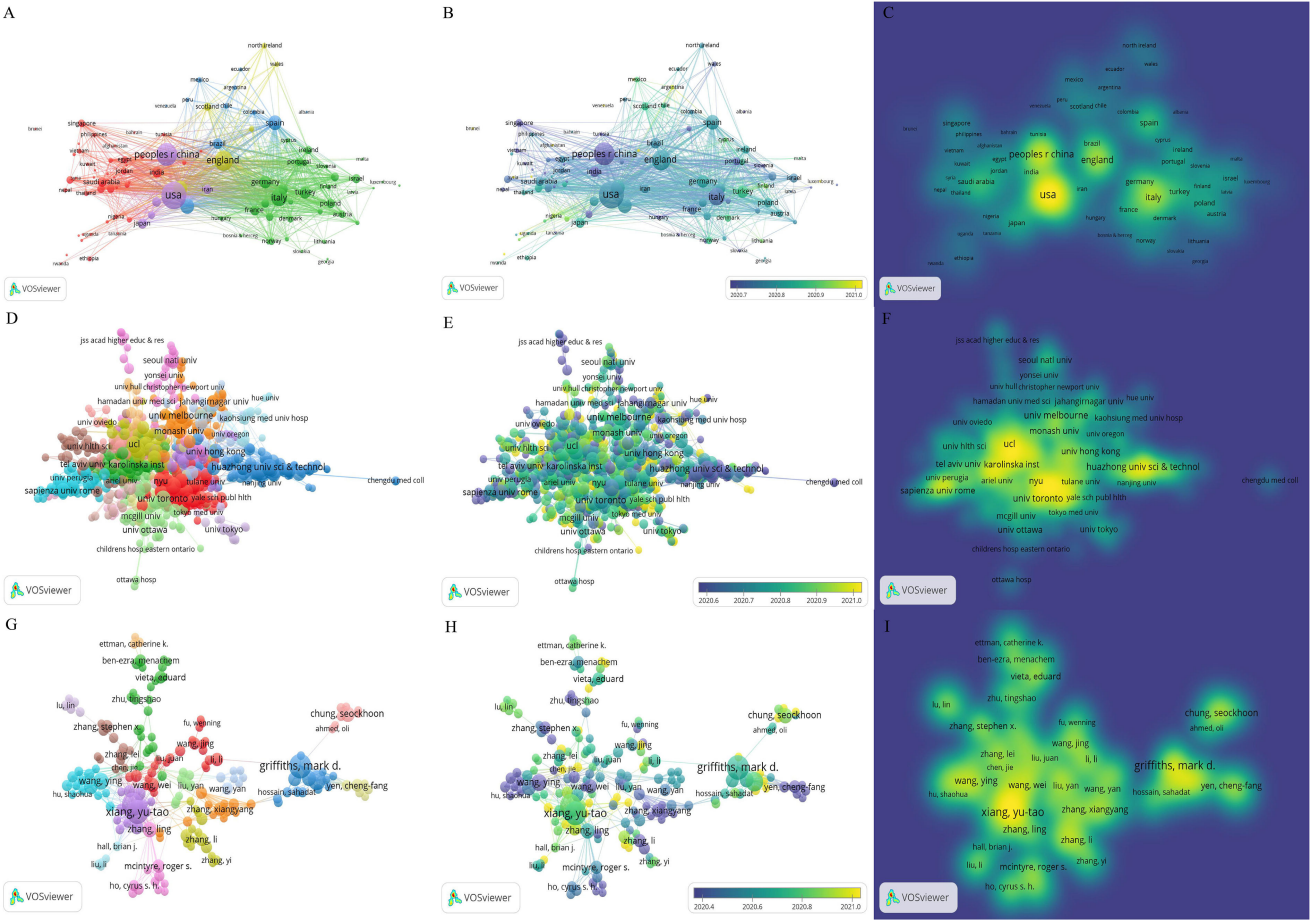
The network map of countries/regions, institutes, and active authors on mental health research related to COVID-19. **(A)** The network visualization map of countries/regions, **(B)** the overlay visualization map of countries/regions, and **(C)** the density visualization map of countries/regions. **(D)** The network visualization map of institutes, **(E)** the overlay visualization map of institutes, and **(F)** the density visualization map of institutes. **(G)** The network visualization map of active authors, **(H)** the overlay visualization map of active authors, and **(I)** the density visualization map of active authors.

The research articles on mental health under COVID-19 were published by 152 countries/regions, and extensive cooperation was noted between them (Figure 2). Table 3 shows the top 10 countries/regions in terms of articles, of which the United States is the most prolific, followed by Mainland China, England, Italy, and Australia.

**Table 3:** Top 10 countries and institutes in the number of articles.

**Rank**	**Country/Region**	**Count**	**Institute**	**Count**
1	United States	2,190	University of London	373
2	Mainland China	1,433	Harvard University	252
3	England	1,090	University of California System	205
4	Italy	800	University College London	197
5	Australia	584	University of Toronto	190
6	Canada	568	King's college London	158
7	Spain	513	Huazhong university science and technology	150
8	Germany	407	Harvard medical school	147
9	Turkey	346	University of Melbourne	129
10	India	271	Sapienza university of Rome	128

Notably, 10,559 institutes participated in mental health research related to COVID-19 (Figure 2). Table 3 lists the top 10 institutes by the number of articles. The 10 institutes account for 21.78% of total articles, among which the University of London has the largest articles (373), followed by Harvard University (252), University of California System (205), and University College London (197).

### Analysis of citations, ESI top papers, and H-index among the top five countries

**Figure 3:**
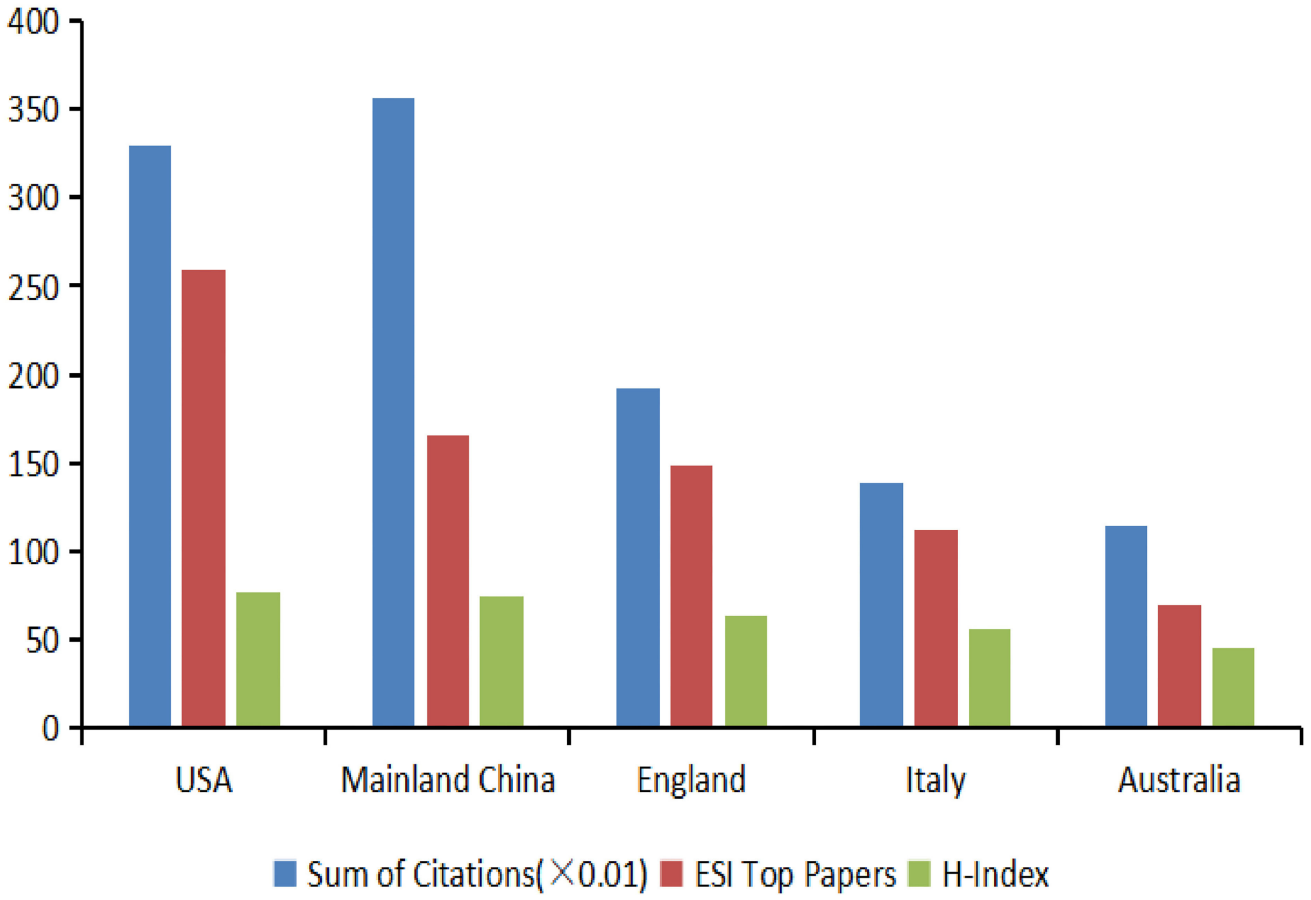
The distribution of citation (×0.01), ESI top papers, and H-index in the top five countries.

A trend analysis of articles and citations was conducted using Excel 2007 (Figure 3). The United States contributed the greatest number of ESI top papers (259) and achieved the highest H-index value (77). China had the greatest number of citations (35,627) and achieved the second-highest H-index value (74). The other three countries were England, Italy, and Australia.

### Distribution of authors

Over 45,010 authors contributed to mental health research related to COVID-19 (Figure 2). Table 4 lists the top 10 authors in the number of articles. Griffiths md (31) ranked first, followed by Xiang yt (30), Cheung t (29), and Lin cy (19).

**Table 4:** The top 10 authors, co-cited authors, and co-cited references.

**Rank**	**Author**	**Count**	**Co-cited Author**	**Count**	**Co-cited Reference**	**Count**
1	Griffiths, Mark D	31	Wang, Chengyu	1,810	Brooks SK, 2020, lancet, v395, p912, doi 10.1016/s0140-6736(20)30460-8	1,335
2	Xiang, Yu-Tao	30	Brooks, Samantha K	1,645	Lai JB, 2020, jama netw open, v3, doi 10.1001/jamanetworkopen.2020.3976	981
3	Cheung, Teris	29	kroenke, k	1,127	Wang CY, 2020, int j env res pub he, v17, doi 10.3390/ijerph17051729	966
4	Lin, Chung-Ying	21	Lai, Jianbo	981	Spitzer RL, 2006, arch intern med, v166, p1092, doi 10.1001/archinte.166.10.1092	755
5	Wang, Ying	16	Spitzer, Robert L	880	Holmes EA, 2020, lancet psychiat, v7, p547, doi 10.1016/s2215-0366(20)30168-1	707
6	Chung, Seockhoon	14	Holmes, Emily A	735	Qiu JY, 2020, gen psychiat, v33, doi 10.1136/gpsych-2020-100213	588
7	Pakpour, Amir H	14	Qiu, Jianyin	595	Kroenke K, 2001, j gen intern med, v16, p606, doi 10.1046/j.1525-1497.2001.016009606.x	537
8	Zhang, Ling	14	Xiang, Yutao	589	Cao WJ, 2020, psychiat res, v287, doi 10.1016/j.psychres.2020.112934	511
9	Liu, Zhongchun	13	Cohen, Sandy	581	Xiang YT, 2020, lancet psychiat, v7, p228, doi 10.1016/s2215-0366(20)30046-8	467
10	Mamun, Mohammed a	12	Kessler, Ronald C	519	Huang YE, 2020, psychiat res, v288, doi 10.1016/j.psychres.2020.112954	459

**Figure 4:**
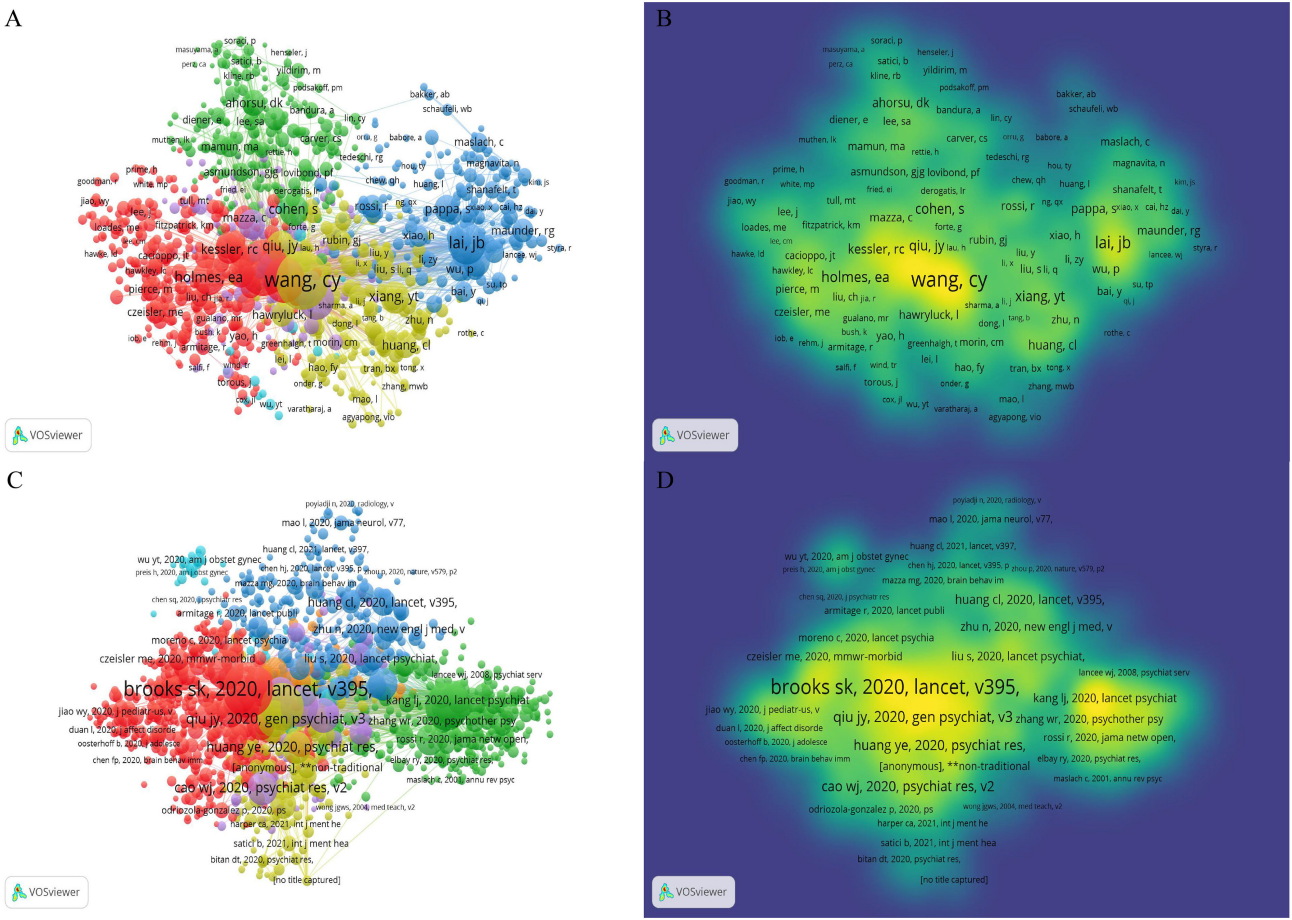
The co-citation map of authors and references on mental health research related to COVID-19. **(A)** The network visualization map of co-cited authors and **(B)** the density visualization map of co-cited authors. **(C)** The network visualization map of co-cited references and **(D)** the density visualization map of co-cited references.

VOSviewer analyzed the information of author citations and visualized it in a co-citation network (Figure 4). Among the top 10 co-cited authors (Table 4), Wang Cy (1,810) ranked first, followed by Brooks sk (1,645), Kroenke k (1,127), Lai jb (981), and Spitzer rl (880).

### Analysis of co-cited references

VOSviewer was used to analyze the co-citation of references. In Figure 4B, the map of density visualization showed that the papers from authors such as Brooks sk, Qiu jy, Huang ye, Kang lj, and Zhang wr have the highest co-citation intensity, suggesting that their research results serve as a good medium in this academic field. In the early stage of the outbreak, people often suffered from anxiety, depression, and panic disorder in psychological problems, and insomnia was often accompanied by prolonged exposure to this state (9, 10). Apart from those who need to be quarantined (11), medical health workers are the most concerned (12, 13). Table 4 lists the top 10 co-cited references.

### Analysis of keywords

**Figure 5:**
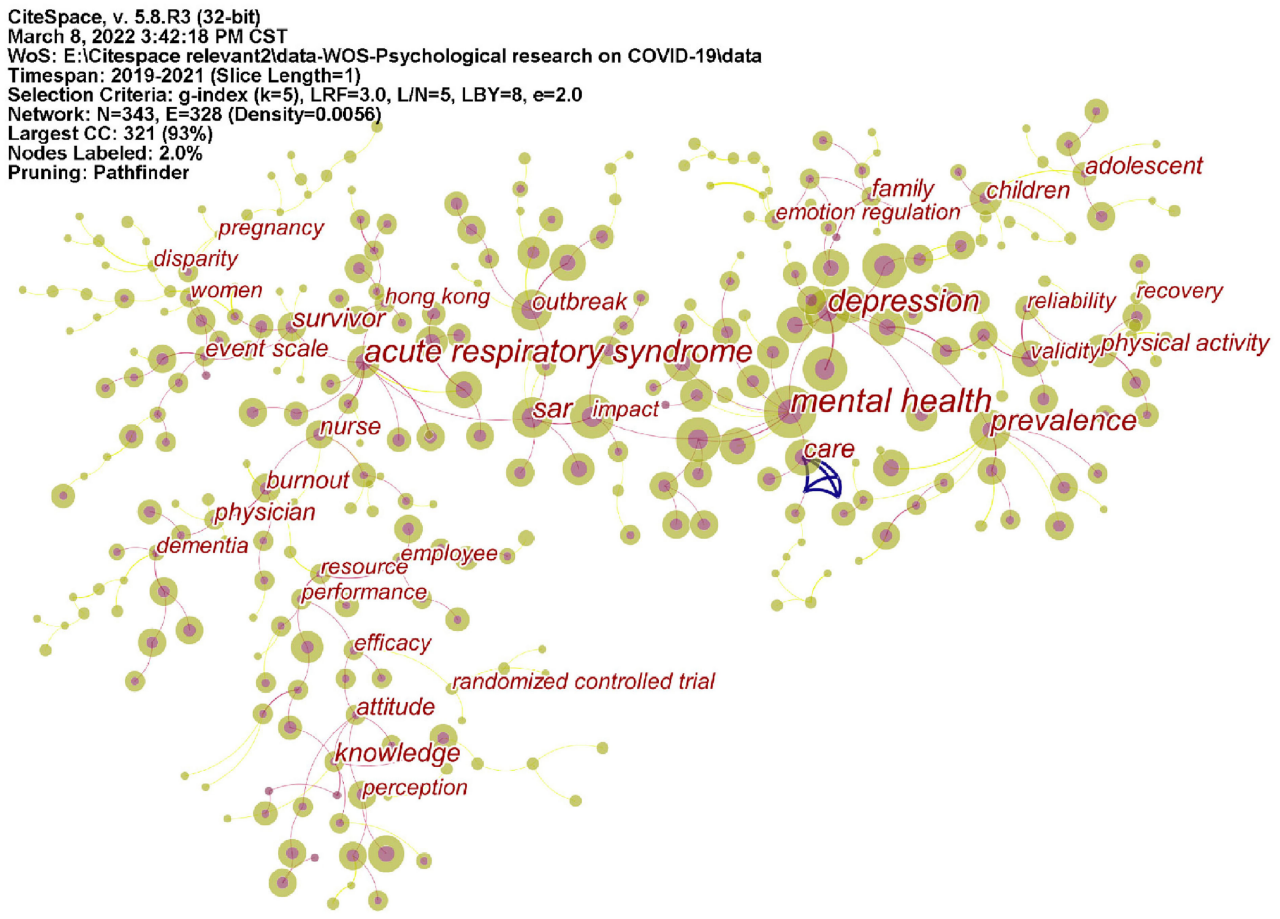
Keywords co-occurrence map of mental health research related to COVID-19.

**Figure 6:**
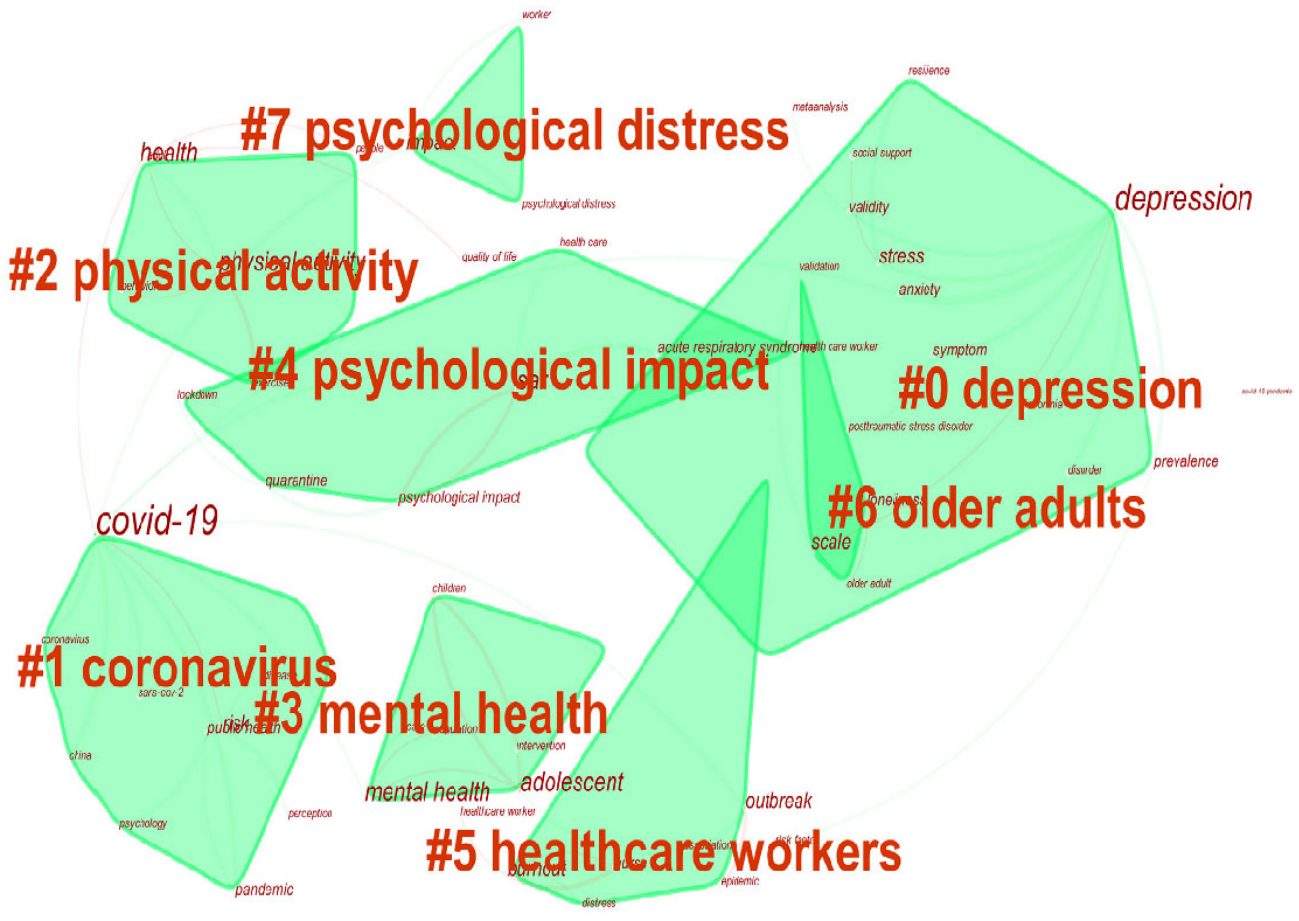
The keyword cluster map for articles on mental health research related to COVID-19.

**Figure 7:**
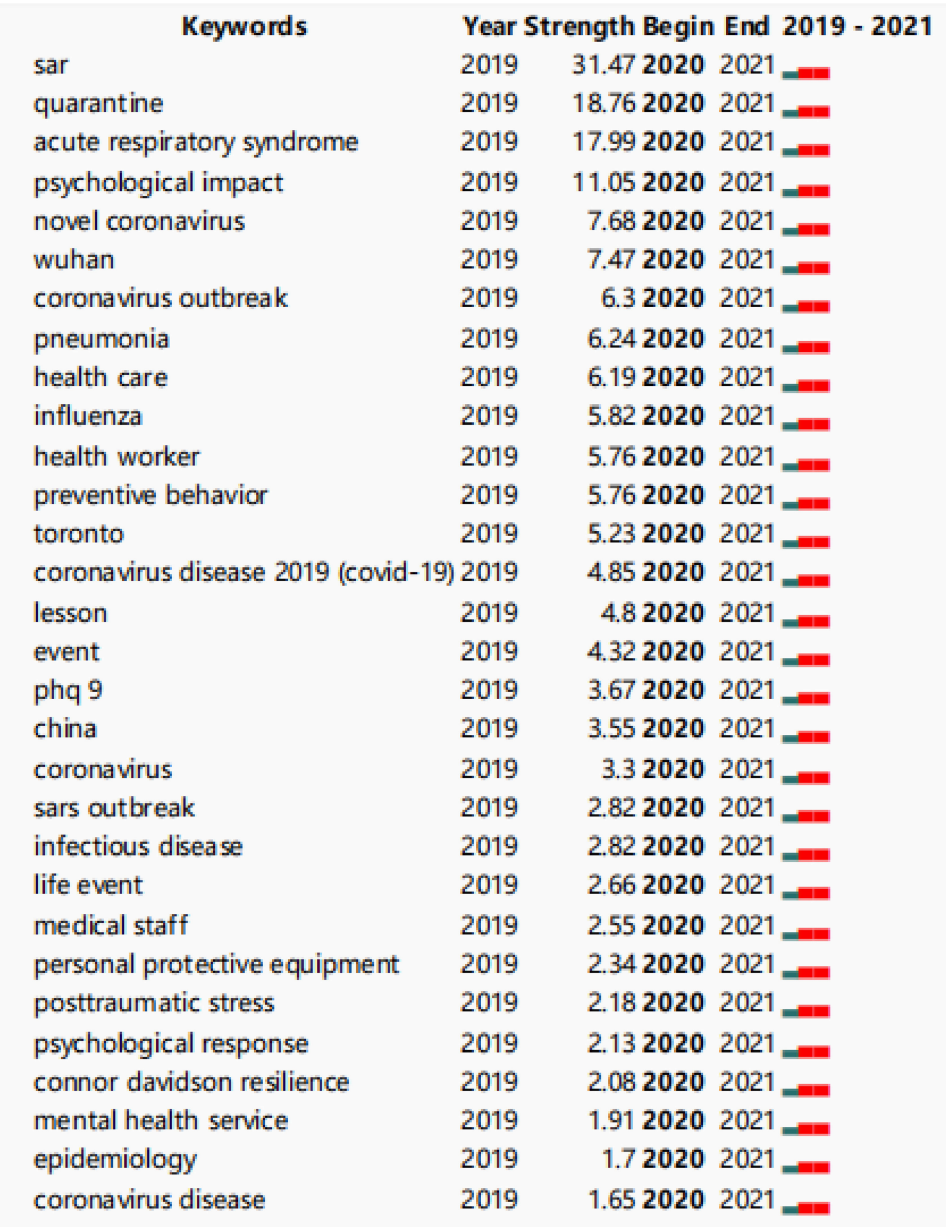
Top 30 keywords with strongest citation bursts.

Map of keywords co-occurrence resulted in 343 nodes and 328 links (Figure 5). As shown in the upper left corner of the picture, the modularity Q was 0.8924 (>0.5), and the mean Silhouette was 0.5368 (>0.5), indicating that the homogeneity of clusters was acceptable (5). In this network (Figure 6), eight cluster labels were listed: #0 depression, #1 coronavirus, #2 physical activity, #3 mental health, #4 psychological impact, #5 healthcare workers, #6 old adults, and #7 psychological distress. Using the burst method to detect keywords, 30 words were obtained (Figure 7).

## Discussion

### General information

With the normalization of the epidemic, vaccination, and the establishment of herd immunity, the post-pandemic era has gradually become widely accepted from the previous unease. In the past 2 years, papers on mental health research related to COVID-19 have shown explosive growth, so it is necessary to systematically summarize and display them in the form of a visual map. Among the top 10 contributive countries/regions, the United States and Mainland China occupied the leading position, accounting for 40.91%. While from the distribution of institutes, Harvard University (USA), University of California System (USA), UCL (UK), University of Toronto (Canada), and King's College London (UK) are the institutions with the most research in this field. Although the number of articles in mainland China was large, high-impact institutions have yet to emerge. In terms of the distribution of authors, Griffiths, mark d ranked first in 31 articles. According to Price's Law (14), the minimum number of articles for core authors *N* = 0.749$\sqrt[{\;}]{M\max}$ (*M*max is the articles of the most prolific authors) and calculated *N*≈4. In terms of the number of articles published, 968 authors have published more than 4, accounting for 2.15% (<50%), indicating that the core author team in this research field has not yet been formed.

### Research hotspots and frontiers

#### Anxiety and depression are common psychological distress

Anxiety or depression can happen to everyone, and for COVID-19 survivors, negative emotions stem from quarantine, stigma, and uncertainty of viral disease progression (15, 16). Studies have shown that baseline systemic immune-inflammation index (SII) reflects positively associated with the score of anxiety and depression at follow-up (17). Levels of CRP, a peripheral inflammatory indicator, correlated positively with the PHQ-9 total score of patients who presented symptoms of depression (16). Compared with infected people, general populations are more derived from panic about the uncertainty of the epidemic, acquaintances or family members are infected, and young people who had to work outside their domicile presented higher levels of anxiety and stress (18).

#### Insomnia and PTSD are common disease syndrome under the epidemic

The incidence of insomnia is higher than that of anxiety and depression in both medical healthcare workers and general populations (19, 20), and someone also accompanied by obsessive-compulsive symptoms (13). PTSD is a serious but treatable mental disorder that occurs after a life-threatening traumatic event, and people show symptoms of traumatic reexperience, hypervigilance, avoidance, and numbness, which usually occur 3 months to several years after the trauma (21, 22). In terms of gender, women are more susceptible, such as pregnant women (23) and nurses (24). A 3-month follow-up record of COVID-19 survivors shows that previous psychiatric diagnosis and obesity were risk factors for developing PTSD (25).

#### The GAD-7 and PHQ-9 scales are most used in psychological assessment

The GAD-7 scale is developed for generalized anxiety disorders (GADs), there are seven problems faced in the past 2 weeks, and each problem is divided into 0–3 points according to the degree of severity. The full score of the GAD-7 scale is 21 points, 0–4 points indicate minimal, 5–9 points are mild, 10–14 points are moderate, and ≥15 points are severe (26). GAD-7 scale is the most commonly used in clinical practice due to its high sensitivity (89%) and specificity (82%) and is widely recognized in the industry. The PHQ-9 scale is the 9-item depression module from the full Patient Health Questionnaire (PHQ) (27), and each problem is divided into 0–3 points according to the degree of severity. The full score of the PHQ-9 scale is 27 points, 0–4 points indicate minimal, 5–9 points are mild, 10–14 points are moderate, 15–19 points are moderately severe, and ≥20 points are severe. PHQ-9 score ≥10 had a sensitivity of 88% and a specificity of 88% for major depression.

#### Healthcare workers, older adults, and college students are high-risk groups prone to psychological problems

In addition to physicians and nurses, healthcare workers also include patient care technicians, respiratory therapists, mental health therapists, etc. In the face of clinical uncertainty, in addition to feeling mental stress, they also have a fear of the unknown, perceived social stigma, and workplace safety concerns (28). Social and family support can effectively alleviate these negative emotions. Older adults who lack social activities and long periods at home are more likely to feel alienated from their families. Unlike anxiety among younger adults, they are more likely to report feelings of loneliness and lack of happiness (29, 30). Additionally, for those elderly who lack access to medical care, this sentiment is even more pronounced (31). For college students, the development of online courses has changed the learning mode that students are familiar with. Economic effects, effects on daily life, and delays in academic activities are positively associated with anxiety symptoms (32). Likewise, insomnia, alcohol use, and gaming behavior will appear (33, 34).

### Strengths and limitations

This study used visualization software to analyze the mental health research under COVID-19 and intuitively showed the research hot spots that scholars around the world have paid attention to in the past 2 years. However, there are also some deficiencies in this study. First, considering the applicability of software to the database, this study only included WOSSC data, and a small part of the data may be omitted. Second, the number of articles on mental health in COVID-19 is increasing, but this study did not assess the quality of the literature, which we need to improve in the future.

## Conclusion

In this study, we summarized the mental health research papers under the epidemic in detail and displayed the cooperation of countries/institutions/authors, co-cited authors/references, and keyword analysis that are also listed through visualization software. Through the maps, we concluded that the most common psychological problems of people during the epidemic are anxiety and depression. Insomnia and PTSD need to be solved under the normalization of the epidemic. GAD-7 and PHQ-9 scales are the most convenient and effective for screening anxiety and depression. Healthcare workers, older adults, and college students should be concerned, and social and family support is essential. Finally, it is worth mentioning that most of the psychological investigations were conducted in a certain area, and most of the psychological investigations were conducted during the outbreak period, but the psychological research on the post-epidemic period was not durable enough. It is suggested that countries and institutions need to further strengthen cooperation, and it is necessary to conduct more long-term follow-ups of patients who have recovered from COVID-19.

## Data availability statement

The original contributions presented in the study are included in the article/Supplementary material, further inquiries can be directed to the corresponding author.

## Ethics statement

Ethical review and approval was not required for the study on human participants in accordance with the local legislation and institutional requirements. Written informed consent from the participants' legal guardian/next of kin was not required to participate in this study in accordance with the national legislation and the institutional requirements.

## Author contributions

RZ and ZW conceived and designed the experiments, performed the experiments, analyzed the data, authored or reviewed drafts of the paper, and approved the final draft. XiaL performed the experiments, analyzed the data, prepared figures and/or tables, and approved the final draft. JX performed the experiments, prepared figures and/or tables, and approved the final draft. XinL and ZW conceived and designed the experiments. ZW authored and approved the final draft. All authors contributed to the article and approved the submitted version.

## Conflict of interest

The authors declare that the research was conducted in the absence of any commercial or financial relationships that could be construed as a potential conflict of interest.

## Publisher's note

All claims expressed in this article are solely those of the authors and do not necessarily represent those of their affiliated organizations, or those of the publisher, the editors and the reviewers. Any product that may be evaluated in this article, or claim that may be made by its manufacturer, is not guaranteed or endorsed by the publisher.

## References

[1] Lopez Bernal J Andrews N Gower C Gallagher E Simmons R Thelwall S. Effectiveness of Covid-19 vaccines against the B. 16172 (Delta) variant. N Engl J Med. (2021) 385:585–94. 10.1056/NEJMoa2108891 34289274 PMC8314739

[2] Torjesen I. Covid-19: Omicron may be more transmissible than other variants and partly resistant to existing vaccines, scientists fear. BMJ. (2021) 375:n2943. 10.1136/bmj.n2943 34845008

[3] Shultz JM Baingana F Neria Y. The 2014 Ebola outbreak and mental health: current status and recommended response. JAMA. (2015) 313:567–8. 10.1001/jama.2014.17934 25532102

[4] van Eck NJ Waltman L. Software survey: VOSviewer, a computer program for bibliometric mapping. Scientometrics. (2010) 84:523–38. 10.1007/s11192-009-0146-3 20585380 PMC2883932

[5] Chen C. CiteSpace II: Detecting and visualizing emerging trends and transient patterns in scientific literature %J. J Am Soc Inform Sci Technol. (2006) 57:317. 10.1002/asi.20317

[6] Chen C. Searching for intellectual turning points: progressive knowledge domain visualization. Proc Natl Acad Sci U S A. (2004) 101(Suppl 1):5303–10. 10.1073/pnas.0307513100 14724295 PMC387312

[7] Chen C Hu Z Liu S Tseng H. Emerging trends in regenerative medicine: a scientometric analysis in CiteSpace. Expert Opin Biol Ther. (2012) 12:593–608. 10.1517/14712598.2012.674507 22443895

[8] Chen CM Leydesdorff L. Patterns of connections and movements in dual-map overlays: a new method of publication portfolio analysis. J Assoc Inform Sci Techno. (2014) 65:334–51. 10.1002/asi.22968

[9] Qiu J Shen B Zhao M Wang Z Xie B Xu Y. nationwide survey of psychological distress among Chinese people in the COVID-19 epidemic: implications and policy recommendations. Gen Psychiatr. (2020) 33:e100213. 10.1136/gpsych-2020-100213 32215365 PMC7061893

[10] Huang Y Zhao N. Generalized anxiety disorder, depressive symptoms and sleep quality during COVID-19 outbreak in China: a web-based cross-sectional survey. Psychiatry Res. (2020) 288:112954. 10.1016/j.psychres.2020.112954 32325383 PMC7152913

[11] Brooks SK Webster RK Smith LE Woodland L Wessely S Greenberg N. The psychological impact of quarantine and how to reduce it: rapid review of the evidence. Lancet. (2020) 395:912–20. 10.1016/S0140-6736(20)30460-8 32112714 PMC7158942

[12] Kang L Li Y Hu S Chen M Yang C Yang BX. The mental health of medical workers in Wuhan, China dealing with the 2019 novel coronavirus. Lancet Psychiatry. (2020) 7:e14. 10.1016/S2215-0366(20)30047-X 32035030 PMC7129673

[13] Zhang W-R Wang K Yin L Zhao W-F Xue Q Peng M. Mental health and psychosocial problems of medical health workers during the COVID-19 epidemic in China. Psychother Psychosom. (2020) 89:242–50. 10.1159/000507639 32272480 PMC7206349

[14] Price DJ. Little Science, Big Science. Columbia: Columbia University Press (1963). 10.7312/pric91844

[15] Benke C Autenrieth LK Asselmann E Pané-Farré CA. Lockdown, quarantine measures, and social distancing: associations with depression, anxiety and distress at the beginning of the COVID-19 pandemic among adults from Germany. Psychiatry Res. (2020) 293:113462. 10.1016/j.psychres.2020.113462 32987222 PMC7500345

[16] Guo Q Zheng Y Shi J Wang J Li G Li C. Immediate psychological distress in quarantined patients with COVID-19 and its association with peripheral inflammation: a mixed-method study. Brain Behav Immun. (2020) 88:17–27. 10.1016/j.bbi.2020.05.038 32416290 PMC7235603

[17] Mazza MG De Lorenzo R Conte C Poletti S Vai B Bollettini I. Anxiety and depression in COVID-19 survivors: role of inflammatory and clinical predictors. Brain Behav Immun. (2020) 89:594–600. 10.1016/j.bbi.2020.07.037 32738287 PMC7390748

[18] Mazza C Ricci E Biondi S Colasanti M Ferracuti S Napoli C. A Nationwide survey of psychological distress among italian people during the COVID-19 pandemic: immediate psychological responses and associated factors. Int J Environ Res Public Health. (2020) 17:3165. 10.3390/ijerph17093165 32370116 PMC7246819

[19] Pappa S Ntella V Giannakas T Giannakoulis VG Papoutsi E Katsaounou P. Prevalence of depression, anxiety, and insomnia among healthcare workers during the COVID-19 pandemic: a systematic review and meta-analysis. Brain Behav Immun. (2020) 88:901–7. 10.1016/j.bbi.2020.05.026 32437915 PMC7206431

[20] Cénat JM Blais-Rochette C Kokou-Kpolou CK Noorishad P-G Mukunzi JN McIntee S-E. Prevalence of symptoms of depression, anxiety, insomnia, posttraumatic stress disorder, and psychological distress among populations affected by the COVID-19 pandemic: a systematic review and meta-analysis. Psychiatry Res. (2021) 295:113599. 10.1016/j.psychres.2020.113599 33285346 PMC7689353

[21] Kirkpatrick HA Heller GM. Post-traumatic stress disorder: theory and treatment update. Int J Psychiatry Med. (2014) 47:337–46. 10.2190/PM.47.4.h 25084856

[22] Yehuda R. Post-traumatic stress disorder. N Engl J Med. (2002) 346:108–14. 10.1056/NEJMra012941 11784878

[23] Kara P Nazik E Nazik H Özer D. Post-traumatic stress disorder and affecting factors in pregnant women in the COVID-19 pandemic. Psychiatr Danub. (2021) 33:231–9. 10.24869/psyd.2021.231 34185755

[24] Jiang H Huang N Tian W Shi S Yang G Pu H. Factors associated with post-traumatic stress disorder among nurses during COVID-19. Front Psychol. (2022) 13:745158. 10.3389/fpsyg.2022.745158 35173657 PMC8841878

[25] Tarsitani L Vassalini P Koukopoulos A Borrazzo C Alessi F Di Nicolantonio C. Post-traumatic stress disorder among COVID-19 survivors at 3-month follow-up after hospital discharge. J Gen Intern Med. (2021) 36:1702–7. 10.1007/s11606-021-06731-7 33782888 PMC8007055

[26] Spitzer RL Kroenke K Williams JBW Löwe BA. brief measure for assessing generalized anxiety disorder: the GAD-7. Arch Intern Med. (2006) 166:1092–7. 10.1001/archinte.166.10.1092 16717171

[27] Kroenke K Spitzer RL Williams JB. The PHQ-9: validity of a brief depression severity measure. J Gen Intern Med. (2001) 16:606–13. 10.1046/j.1525-1497.2001.016009606.x 11556941 PMC1495268

[28] Van Wert MJ Gandhi S Gupta I Singh A Eid SM Haroon Burhanullah M. Healthcare worker mental health after the initial peak of the COVID-19 pandemic: a us medical center cross-sectional survey. J Gen Intern Med. (2022). 10.1007/s11606-021-07251-0 PMC8734540 34993856

[29] López J Perez-Rojo G Noriega C Carretero I Velasco C Martinez-Huertas JA. Psychological well-being among older adults during the COVID-19 outbreak: a comparative study of the young-old and the old-old adults. Int Psychogeriatr. (2020) 32:1365–70. 10.1017/S1041610220000964 32438934 PMC7324658

[30] Vahia IV Jeste DV Reynolds CF. Older adults and the mental health effects of COVID-19. JAMA. (2020) 324:2253–4. 10.1001/jama.2020.21753 33216114

[31] Barber SJ Kim H. COVID-19 Worries and behavior changes in older and younger men and women. J Gerontol B Psychol Sci Soc Sci. (2021) 76:e17–23. 10.1093/geronb/gbaa068 32427341 PMC7313781

[32] Cao W Fang Z Hou G Han M Xu X Dong J. The psychological impact of the COVID-19 epidemic on college students in China. Psychiatry Res. (2020) 287:112934. 10.1016/j.psychres.2020.112934 32229390 PMC7102633

[33] Balhara YPS Kattula D Singh S Chukkali S Bhargava R. Impact of lockdown following COVID-19 on the gaming behavior of college students. Indian J Public Health. (2020) 64(Supplement):S172–S6. 10.4103/ijph.IJPH_465_20 32496250

[34] Charles NE Strong SJ Burns LC Bullerjahn MR Serafine KM. Increased mood disorder symptoms, perceived stress, and alcohol use among college students during the COVID-19 pandemic. Psychiatry Res. (2021) 296:113706. 10.1016/j.psychres.2021.113706 33482422 PMC7781902

